# Life and death of lymphocytes: a role in immunesenescence

**DOI:** 10.1186/1742-4933-2-12

**Published:** 2005-08-23

**Authors:** Sudhir Gupta, Houfen Su, Ruifen Bi, Sudhanshu Agrawal, Sastry Gollapudi

**Affiliations:** 1 Laboratories of Cellular and Molecular Immunology and Molecular Biology, Division of Basic and Clinical Immunology, University of California, Irvine, California 92697, USA

**Keywords:** Tumor necrosis factor, Fas, mitochondria, aging, memory T cells, Activation-induced cell death

## Abstract

Human aging is associated with progressive decline in immune functions, increased frequency of infections. Among immune functions, a decline in T cell functions during aging predominates. In this review, we will discuss the molecular signaling in two major pathways of apoptosis, namely death receptor pathway and mitochondrial pathway, and their alterations in both T and B lymphocytes in human aging with a special emphasis on naïve and different memory subsets of CD8+ T cells. We will also discuss a possible role of lymphocyte apoptosis in immune senescence.

## Introduction

**Figure 1:**
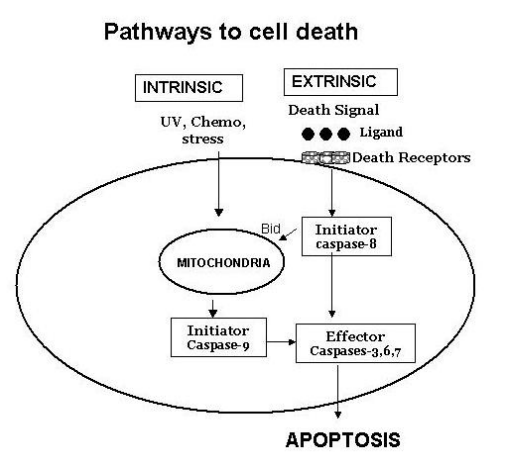
Two distinct pathways of apoptosis. Death receptor pathway and mitochondrial pathway use distinct initiator caspases but common effector caspases. Death receptor and mitochondrial pathways are linked via Bcl-2 family protein Bid.

Apoptosis is a physiological form of cell death, which plays an important role in embryogenesis, metamorphosis, cellular homeostasis, tissue atrophy and removal of tumor and mutated cells. In the immune system, apoptosis appears to play a crucial role in selection of T cell repertoire in the thymus, deletion of self-reactive T lymphocytes and B lymphocytes, regulation of immunological memory, deletion of effector T cells following an effective immune response, and in the cytotoxicity of target cells by CD8+ T cells and natural killer cells [1-3]. There are two major signaling pathways of apoptosis (Figure 1), the death receptor pathway (extrinsic pathway) and intrinsic pathway the mitochondrial pathway [4-11]. The apoptosis via both pathways is mediated by the activation of a series of cysteine proteases, the caspases. Caspases act as molecular chainsaw, which cleave a number of cytoplasmic and nuclear substrates to induce characteristic of apoptosis. Although both pathways of apoptosis involve activation of common effector or executioner caspases, they differ in the activation of apical or initiator caspases. Caspases are present in inactive form as prozymes. Apical caspases are autolytically activated by homodimerization without undergoing cleavage, whereas executioner caspases are activated via cleavage of their prodomain by apical caspases. Both pathways also recruit different adaptor molecules. In this article we will review differential sensitivity of various T lymphocyte subpopulations to apoptosis and their changes during aging and the role of subsets of T cells that are sensitive or resistant to apoptosis in immune senescence. A role of apoptosis in B lymphocytes in aging will also be briefly discussed.

## Death Receptor Pathway of Apoptosis

Death receptors belong to a large family of tumor necrosis factor receptors (TNFRs) and nerve growth factor receptors (NGFRs). Following interaction with death receptor ligand the cytoplasmic death domain (DD) of death receptor undergo trimerization, which leads to recruitment of a set of adaptor proteins and proximal caspase to form a death-inducing signaling complex (DISC). DISC serves as a platform for the activation of downstream caspases and apoptosis. In the DISC, initiator caspases undergo activation by homodimerization and without cleavage. Activated initiator caspases cleave effector caspases, which cleaves a number of cytoplasmic and nuclear substrates to induce apoptosis. We will discuss three distinct forms of death receptor-mediated apoptosis, which have been studied in human aging.

### Activation-induced cell death

The activation-induced cell death (AICD), in which activation of T cells occurs through proper engagement of T cell receptors (TCRs) by specific antigen bound to MHC molecule and influenced by antigen concentration, and co-stimulatory signals. AICD plays an essential role in both central and peripheral deletion (clonal deletion) events involved in tolerance and homeostasis [12]. The AICD appears to be mediated primarily by an interaction between CD95 and CD95L [13-15]. In the AICD, cells are initially activated by anti-CD3 for 5 days and then re-stimulated with anti-CD3 to induce apoptosis, whereas in CD95-mediated apoptosis cells are first activated with anti-CD3 and cultured in IL-2 containing medium followed activation with anti-CD95 antibody or CD95L to induce apoptosis. AICD occurs only in the cells of the immune system, whereas CD95-mediated apoptosis may occur in any cell type. CD95-CD95L interaction is essential for AICD in mature T cells *in vitro *[16,17] and *in vivo *for peripheral T cell deletion [18,19].

### CD95-mediated apoptosis

**Figure 2:**
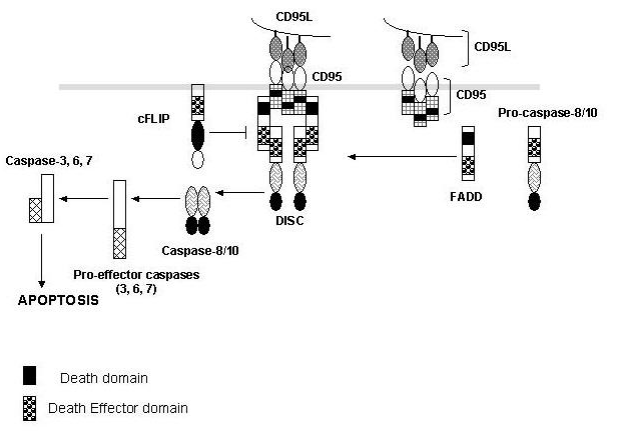
CD95-mediated Apoptosis. CD95 upon ligation with CD95 ligand (CD95L) undergo trimerization resulting in the recruitment of fas-associated death domain (FADD) and procaspase-8 to form death-inducing signaling complex (DISC). Procaspase-8 is autolytically activated by homodimerization and released from the DISC into the cytosol, where it cleaves and activate effector caspases to induce apoptosis.

CD95 is a member of type I transmembrane receptors that is constitutively expressed on lymphocytes; however, CD95L, a type II transmembrane protein is lacking from resting lymphocytes and is transcrptionally regulated and induced upon activation of lymphocytes. The steps of CD95-mediated apoptosis signaling pathway are shown in Figure 2. Upon ligation with soluble CD95L or anti-CD95 monoclonal antibodies CD95 undergoes trimerization. Cytoplasmic DD of CD95 recruits an adapter protein, the fas-associated death domain (FADD), which contain a death effector domain (DED). FADD then recruits and through homologous and protein-protein interaction binds to procaspse-8 (Flice) to form a death-inducing signaling complex (DISC), which serves as a platform to initiate enzymatic activation of apoptotic pathway. Procaspase-8 is autolytically activated by homdimerization to generate active caspase-8, which is released from the DISC into the cytoplasm where it cleaves effector caspases (caspase-3, caspase-6, caspase-7) to generate active effector caspases. Active effector caspases in turn cleave a number of substrates to elicit characteristic morphological and biochemical features of apoptosis. This classical pathway occurs in so called type I cells [20]. In type II cells, procaspases-8 levels are very low and therefore caspase cascade is amplified via mitochondrial pathway. Caspase-8 cleaves the Bid, a Bcl-2 family member, to produce a truncated form of Bid (tBid), which then translocates from the cytoplasm to the mitochondria and exerts is proapoptotic effect by inhibiting Bcl-2/Bcl-x_L _resulting in the release of cytochrome c, activation of intiator caspase-9 and then of effector caspases resulting in apoptosis [21].

### TNFR-mediated Apoptosis

TNF-α is a plieotropic cytokine, which exerts its biological activity by binding to both type I and type II receptors (TNFR-I and TNFR-II) and activating several signaling pathways [2-7,22-25]. TNFRs belong to a family of TNFRs/NGFRs [26]. Both TNFRs receptors contain one to five cysteine-rich repeats in their extracellular domains; however differ in their cytoplasmic domain. TNFR-1 contains DD whereas TNFR-2 lacks DD. Therefore, TNFR-I signals both cell survival and cell death signals; whereas TNFR-II primarily mediates primarily a cell survival signals. However, recent data suggest that TNFR-II may also participate in apoptosis and may potentiate death signal mediated by TNFR-I. Both cell survival and cell death signals mediated by TNFRs require distinct sets of adapter and other downstream signaling molecules.

**Figure 3:**
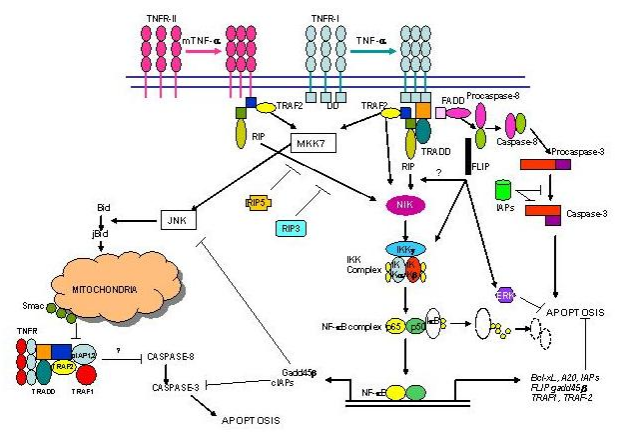
TNFR-mediated apoptosis. TNFR-I upon interaction with TNF-α undergo trimerization and recruitment of TNFR-associated death domain (TRADD), TNFR-associated factor 2 (TRAF-2) and receptor-interacting protein (RIP) to form complex I. This complex activates NF-κB via phosphorylation of IKK and IκB. NF-κB inhibits apoptosis by inducing a number of ant-apoptotic molecules (Bcl-xL, cIAPs, FLIP, Gadd45β, A20). After internalization of TNFR-I, TRADD, TRAF 2, and RIP are dissociated from the complex and FADD and caspase-8 are recruited (Complex II) to induce apoptosis. TRAF2 also activate JNK and sustanin activation of JNK induces apoptosis via selective release of Smac from the mitochondria.

Steps of TNFR-mediated signaling are shown in Figure 3. TNFR-I undergo trimerization of its receptor death domains, which in turn recruit an adaptor protein, TNFR-associated death domain (TRADD). TRADD then may recruit another adapter molecule, the Fas-associated death domain (FADD). FADD then recruits procaspase-8, which is autolytically activated and then induces apoptosis via activation of effector caspases. TRADD may recruit distinct sets of adapter proteins, TRAF-2 (TNF-R-associated factor-2) and receptor interactive protein (RIP). TRAF-2 and RIP stimulate pathways leading to activation NFκB. Studies in mice and humans have shown that NF-κB is a repressor of apoptosis [27-31]. However, until recently it was unclear how NF-κB activation by TNF-α could inhibit initiator caspase activation through the same receptor (TNFR-I).

Recently, Jurg Tschopp's group has proposed a two complex model based upon their experimental findings that TNFR-I signaling involve assembly of two distinct complexes that sequentially activate NF-κB and caspases [32]. In this model, the binding of TNF to TNFR-I results in the formation (within minutes) of signaling complex I. This complex contains TNFR-I, TRADD, RIP, and TRAF-2. Signaling complex I leads to activation of NF-κB via recruitment of (IκB kinase) IKK complex and phosphorylation of IκB. The secondary complex is form possibly following TNFR-I internalization (>2 hours following interaction between TNF and TNFR-I) in which TRADD, RIP, and TRAF-2 dissociate from the receptor and recruits FADD and caspase-8 (complex II). In conditions of complex I signaling, which leads to strong NF-κB activation, gene expression of anti-apoptotic proteins is induced and the activation of initiator caspases in complex II is inhibited. In contrast, when complex I signaling results in weak or deficient NF-κB activation, the products of anti-apoptotic gene are not made, and complex II can signal apoptosis via activation of caspases.

A family of TRAFs functions as adaptor molecules for TNFR superfamily members by associating with the intracellular domain of these proteins and subsequently mediating downstream signaling events such as activation of NF-κB. TRAF2 is recruited to TNFR-I signal complex via TRADD and plays a positive role in canonical pathway that activates NF-κB through IKKβ. TRAF2 homodimers as well as TRAF1:TRAF2 heterodimers can associate with TNFR-II that is required for signaling and NF-kB activation [33] TRAF2 also plays a role in TNF-induced activation of JNK via MEKK1 [34]. TRAF2 also ubiquitinates RIP at K63 (without proteasomal degradation) to activate NF-κB. Unlike TNFR-I, TNFR-II binds TRAF2 directly, hence activates IKK and JNK (TRAF-2 is also involved in TNFR-II-mediated activation of NF-κB). TRAF-2 also recruits ancilliary proteins (cIAP1, cIAP2, TRAF1, A20) that modulate signaling though each TNFRs and inhibit apoptosis. cIAP-TRAF2 complex inhibits caspases-8 activation by an unknown mechanism. Simultaneous engagement of both TNFR-I and TNFR-II amplifies TNF-induced apoptosis [35,36]. This correlates with increased TNFR-II-induced degradation of TRAF2. Since TRAF2 recruits cIAPs to TNFR-I, its degradation by TNFR-II may facilitate apoptosis by dissociation of cIAP from TRAF-2-cIAP complex and therefore allowing activation of caspase-8. In addition, TRAF2 degradation may also attenuate TNFR-I-mediated activation of NF-κB and promote apoptosis.

Receptor-interactive protein (RIP) is serine/threonine kinase, which is a component of TNFR-I signaling complex and is required for TNFR-I-mediated NF-κB activation [37-39]. RIP contains three domains, including an N-terminal kinase domain, an intermediate domain (which interact with the RING finger domain of TRAF-2) and an N-terminal DD. RIP interacts with TRADD through their respective DDs via protein-protein interaction. RIP family consist of five members, including RIP2, RIP3, RIP4, and recently described RIP5 [40-44]. All RIP kinases share significant similarities in their N-terminus kinase domain, but differ in their C-terminus domain. RIP, RIP2 and RIP4 are involved in the activation of NF-κB (42–44); RIP4 is also involved in JNK activation. Recently, it has been reported that RIP3 and RP5 are involved in TNFα-induced apoptosis [40,42]. RIP3 exerts its pro-apoptotic activity by activating caspases and/or by inhibiting RIP- and TNFR-1-induced NF-κB activation.

NF-κB mediates its repressor effect on apoptosis by inducing the expression of a number of anti-apoptotic genes including cIAPs, FLIP, TRAF-1, TRAF-2, Bcl-2, and Bcl-x_L _[30,31,45].

Inhibitor of apoptosis protein (IAP) family proteins, originally identified in the genome of baculovirus, has a key role in the negative regulation of apoptosis [46,47]. The cIAP-1 and cIAP2, two structurally homologous proteins, belong to a family of death inhibitors sharing a motif found in a Baculovirus inhibitor of death. cIAP1 and cIAP2 were initially isolated by their interaction with TRAF-1 and TRAF-2 in the TNF-RII complex. cIAP1 is also recruited to the DISK of TNF-RI by TRAF-2. In addition to cIAP1 and cIAP2, XIAP have a conserved COOH-terminal RING finger, zinc-binding domain [48]. Overexpression of these mammalian IAPs confers resistance to apoptosis. These proteins suppress apoptosis by preventing the activation of procaspases and inhibiting directly the enzyme activity of mature caspases. XIAP is a potent, active site-directed inhibitor of the effector caspases-3. In addition, TRAF-2-IAP complex inhibits caspases-8 activation by an unknown mechanism.

A20, a ring finger protein, was initially characterized as an inhibitor of TNF-α-induced apoptosis [49]. A20 is peculiar because it has dual activity in that it inhibits apoptosis as well as NF-κB activation [50]. These activities of A20 are cell type specific. A20 inhibits NF-κB activation by both deubiquitination (of K63 ubiquitination of RIP) and subsequent K48 ubiquitination for S26 proteasomal degradation of RIP. The fact that the expression of A20 is itself under control of NF-κB suggests that A20 is involved in the negative feed-back regulation of NF-κB activation. In contrast, A20 inhibits apoptosis, at least partially, by binding to TXBP151, which inhibits TNF-α-induced apoptosis. Furthermore, A20 and cIAP interact with a common region in TRAF2 [51]. Therefore, it is possible that A20 releases cIAP from the TRAF2-signaling complex, thereby allowing these proteins to exert their anti-apoptotic effects. Anti-apoptotic activity of A20 is restricted to certain cell type and is associated with decreased activation of caspases-3.

cFLIP is one of the apoptosis regulatory molecules that is induced by NF-κB [52]. FLIP comes in two spliced forms, the c-FLIP_L _and c-FLIPs. c-FLIPs contains two tandem repeat death effector domains (DED) and inhibits procaspase activation in the DISC. In contrast, c-FLIP_L _shares extensively homology with procaspase-8 yet it is enzymatically inactive [53]. In addition to its inhibitory effect on procaspase-8 activation, c-FLIP associates with Raf-1, which activates MEK1 to activate ERK, and with TRAF1 and TRAF2, which lead to NF-κB activation [54].

MAPK may inhibit [55] or promote apoptosis [56] via transient (inhibits apoptosis) or sustained (promotes apoptosis) activation of Janus-like kinase (JNK). Recently, a role of JNK in TNF-induced apoptosis has been explored [57]. JNK activation is required for TNF-induced apoptosis. Deng et al [58] demonstrated that TNF-α-induces apoptosis via sustained activation of JNK, which cleaves Bid, in a caspases-8-independent manner, to yield a unique 21kDa Bid cleaved product (jBid), which is different from caspases-8-dependent cleaved Bid (tBid) of 15kDa. jBid translocates to the mitochondria and preferentially releases Smac/Diablo from the mitochondria, which may disrupt TRAF-2-cIAP1 complex formation and its inhibition on caspases-8 activation. In addition Smac inhibits anti-apoptotic effects of cIAP and XIAP by binding it to them. De Smaele et al [59] identified GADD45β as an inhibitor of JNK activation and inhibitor of TNF-α-induced apoptosis. However, *gadd45β *is the only gene in this family that appears to be regulated by NF-κB and its ectopic expression completely suppresses TNF-α-induced apoptosis. This provides another mechanism via which NF-κB inhibits apoptosis.

Unlike TNF-RI, TNF-RII lack a cytoplasmic DD, instead interaction between TNF-α and TNF-RII results in binding of TRAF1 and TRAF2 to the cytoplasmic portion of TNF-RII. This then recruits the cellular inhibitor of apoptosis proteins cIAP-1 and cIAP-2 [46,51]. However, it has been reported that TNF-RII may also play an important role in the regulation of apoptosis through TNF-RI. Several investigators have reported that TNF-RII potentiates TNF-α-induced apoptosis [60-64] and proposed a number of mechanisms to explain this observation, including TNF-RII serving as high affinity trap of TNF-α that delivers TNF-α to TNF-RI [65], and direct induction or potentiation of apoptosis by the cytoplasmic domain of TNFR II [62,66].

## Mitochondrial Pathway of Apoptosis

**Figure 4:**
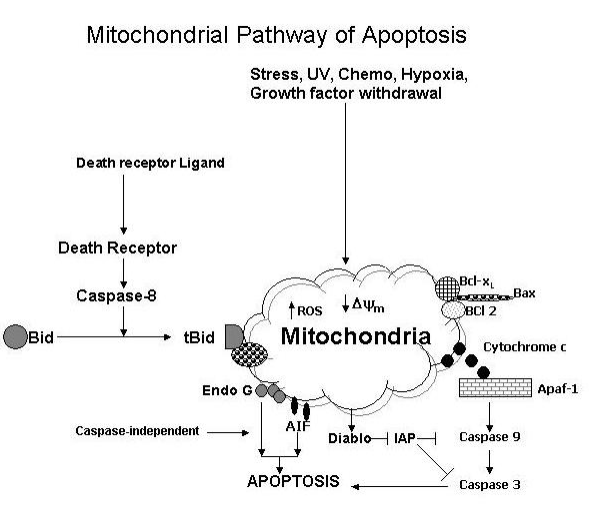
Mitochondrial pathway of apoptosis. See text for details.

Several recent publications have reviewed the subject of mitochondrial pathway of apoptosis [7-11,67]. A number of stimuli, including chemotherapeutic agents, UV radiation, stress molecules (reactive oxygen and reactive nitrogen species) and growth factor withdrawal may mediate apoptosis via mitochondrial pathway In certain cell type mitochondrial pathway may provide an amplifying mechanism for death receptor-mediated apoptosis. Mitochondria contain two well-defined compartments: the matrix, surrounded by the inner membrane (IM), and the intermembrane space, which is surrounded by the outer membrane (OM). The IM contains various molecules, including ATP synthase, electron transport chain, and adenine nucleotide translocator (ANT). Under physiological conditions these molecules allow the respiratory chain to create an electrochemical gradient (membrane potential). The OM contains a voltage-dependent anion channel (VDAC). Bcl-2 is located on the IM and appears to play an important role in the maintenance of mitochondrial membrane potential (ΔΨm). The intermembrane space contains holocytochrome c, certain pro-caspases, adenylate kinase 2, Endo G, Daiblo/Smac, and apoptosis-inducing factor (AIF). The permeabilization of the OM, therefore, results in the release of these molecules into the cytoplasm. IM permeabilization leads to changes in ΔΨm. Once released from the mitochondria, cytochrome c binds to an adapter molecule Apaf-1 (Apoptotic protease-activating factor) in the presence of ATP/dATP and recruits pro-caspase 9 for form apoptosome (Fig. 4). Procaspase-9 is dimerized and activated without undergoing cleavage, and active caspases-9 activates executioner caspases to orchestrate apoptosis.

A number of molecules present in the mitochondrial intermembrane space can promote apoptosis in caspases-independent manner. Htra2/Omi, in addition to its ability to block IAPs, appears to promote caspases-independent apoptosis via its serine protease activity [68,69]. Apoptosis inducing factor (AIF) is a caspases-independent death effector, which upon induction of apoptosis translocates from intermembrane space of the mitochondria to the nucleus where it AIF causes chromatin condensation and large scale DNA fragmentation [70,71]. Endo G, upon its release from mitochondrial intermembrane space, appears to directly mediate nuclear DNA fragmentation in a caspase-independent manner [72].

The mitochondrial membrane permeabilization (MMP) is controlled by a variety of members of the Bcl-2 family [7-11,73]. The Bcl-2 family members are divided into three groups: anti-apoptotic (Bcl-2, Bcl-xL, Mcl-1, Bcl-w, and A1), pro-apoptotic "BH3 only" (Bid, Bim, Bik, Bmf, Bad, Hrk, BNIP3) and pro-apoptotic "BH-123" (Bax, Bak, and Bok) proteins.

Several of the pro-apoptotic members of the Bcl-2 family, including Bax, Bak, Bad, Bid, and Bim, initiate MMP by forming what appears to be a channel. In order to influence their effects, the members of Bcl-2 pro-apoptotic family must dock onto the mitochondrial OM. During apoptosis Bax, which is present in the cytoplasm in a monomer form, is translocated to the mitochondrial membrane to form a dimer or high order oligomers. Bak can also loosely associate with OM. Bim, present in microtubules, also translocates to OM during apoptosis. Bim is a calcium-dependent proapoptotic molecule. Bcl-2 and Bcl-x_L _inhibit cytochrome C release. The phosphorylation of members of the Bcl-2 family rendered them inactive. In response to genotoxic agents, the stress-activated protein kinase (SAPK, also termed c-jun amino-terminal kinase or JNK) translocates to mitochondria and phosphorylates Bcl-x_L_, leading to Bcl-x_L _inactivation and induction of apoptosis.

## Apoptosis in T Lymphocytes in Aging

Apoptosis in lymphocytes in aged humans has been studied primarily via death receptor signaling. Recently we (manuscript submitted) and others [74] have also studied apoptosis in human B lymphocytes.

### Death receptor-induced apoptosis in CD4+ and CD8+ T cells in aging

During human aging (in contrast to mice) there is a progressive T cell lymphopenia, which is shared by both CD4+ and CD8+ T cells [75,76]. Although there has been controversy regarding lymphopenia in aging, our studies were performed in aged subjects from middle class social status, each of them own his/her house, living independently, and were asked to discontinue any anti-oxidants they might be taking for at least one week prior to the study (75). Therefore, our population of seniors does not have any nutritional or extrinsic factors and changes in lymphocyte counts and T cell subsets appear to reflect true changes of aging. Furthermore, many of our subjects were tested on two to three separate occasions. Although the precise mechanism of lymphopenia in aging is unclear, it is likely that decreased bone marrow precursors, decreased thymic output, reduced proliferative potentials and/or increased apoptosis, may contribute to T cell lymphopenia during human aging.

### Activation-induced cell death (AICD) and CD95-mediated apoptosis in CD4+ and CD8+ T cells in aging

Apoptosis of T cells is increased during human aging [77-88]. Phelouzat et al [84,85] and Lechner et al [86] reported that T cells from aged individuals undergo increased AICD as compared to cells from young subjects and increased apoptosis was associated with increased expression of CD95. Potestio et al [87] reported increased spontaneous and AICD in T cells from aged humans and a correlation between increased spontaneous apoptosis and increased CD95 expression; however, we have observed better correlation between spontaneous apoptosis and CD95L expression rather than with CD95 expression [89].

In our study, using different methods to detect apoptosis including propidium iodide and TUNEL assay, Hoechst 33342 staining, and DNA fragmentation by gel electrophoresis, we observed that both CD4+ and CD8+ T cells from aged healthy subjects were more sensitive to anti-CD95-induced apoptosis as compared to young healthy control [77]. Increased apoptosis was associated with increased expression (at protein level) and increased and early activation of both caspase-8 and caspase-3 [90]. Furthermore, both CD4+ and CD8+ T lymphocytes from aged humans display increased expression of CD95 and CD95L. In addition, we observed higher apoptosis in CD4+ T cells as compared to CD8+ T cells. Zeng et al [91] have also observed preferential anti-CD95-induced death of CD4+ T cells.

### TNFR-mediated apoptosis in CD4+ and CD8+ T cells

During aging, TNF-α production is increased [92-98]. We showed that both CD4 and CD8 cells from the elderly were more susceptible to TNF-α-induced apoptosis as compared to young subjects [2,6,7,76,78-82]. Furthermore, increased sensitivity of T cell subsets from aged humans to TNF-α-induced apoptosis was associated with increased and early activation of both caspase-8 and caspase-3. In contrast to our observations, Salvoni et al [99], using freshly isolated T cell subsets and using TNF-α and cyclohexamide to induce apoptosis, observed that aged CD4+ T cells were more resistant to TNF-α-induced-apoptosis as compared to young controls. However, these investigators demonstrated increased susceptibility of aged CD8+ T cells to apoptosis by Annexin V staining. In this study the expression of TNFRs or activation of caspases were not studied. These differences may be due to differential expression of TNFRs. The externalization of posphatidyl serine (which binds to Annexin V) is mediated by scramblase enzyme, which is sensitive to calcium. Therefore, significant changes in intracellular calcium may result in a cell to be positive for Annexin V without undergoing apoptosis; calcium signaling is different among CD4+ and CD8+ T cells and among young and aged T cells (unpublished data). In addition, no data of the effect of cyclohexamide alone or on Annexin V positivity was presented. In our study, we have used a model of *in vivo *activation and no cyclohexamide was used. The sensitivity of T cells to TNF-α-induced apoptosis appears to be age-dependent as cord blood lymphocytes are least sensitive [100] whereas aged T cells are most sensitive to TNF-α-induced apoptosis [78].

We also examined a role of downstream signaling molecules in increased apoptosis in aged T cells. We observed increased expression of TRADD and FADD in lymphocytes from aged subjects both at the level of mRNA and protein [77,78]. However, the expression of RIP both at the mRNA level and the protein level in aged lymphocytes was similar to lymphocytes from young subjects [78].

We have also reported that aged T cell subsets are sensitive to anti-CD95-induced apoptosis [76]. Since, FADD is common conduit for both CD95- and TNFR-mediated apoptosis we examine a role of increased FADD expression on increased apoptosis in aging. T cells from aged humans transfected with dominant negative FADD resulted in decreased TNF-α-induced apoptosis to a level comparable to young T cells, whereas wild type FADD resulted in increased apoptosis in both young and aged T cells albeit to a greater extent in young T cells to a level comparable to aged T cells, establishing a role of increased FADD in increased apoptosis in aged T cells [101].

Furthermore, we investigated whether downregulation of NF-κB activation (an anti-apoptotic signal) may also play a role in increased TNF-α-induced apoptosis. We have observed decreased TNF-α-induced DNA-binding activity of NF-κB in lymphocytes from aged humans as determined by EMSA and recently developed ELISA assay [102]. To further define the molecular mechanism of decreased NF-κB activity, we examined the expression of phosphorylated IKKβ and IκB. T cells from aged humans expressed low levels of phosphorylated IKKβ and Iκ-B. Furthermore, overexpression of IKKβ in aged T cells resulted in an increased phosphorylation of Iκ-B and decreased TNF-α-induced apoptosis in aged T cells to a level comparable to T cells from young subjects. NF-κB mediates its antiapoptotic effect via induction/upregulation of a number of anti-apoptotic genes, including Bcl-2, Bcl-xL, cIAPs, FLIP, and Gadd45β [30,31,45]. We have previously reported that in aging expression of Bcl-2 and cIAP1 is decreased [77,103]. We also showed that overexpressed IKKβ-induced inhibition of increased apoptosis in aged lymphocytes was associated with an upregulation of Bcl-2 and cIAP2 [102]. cIAP2 expression is regulated by NF-κB and therefore decreased cIAP2 in aging would be consistent with decreased NF-κB activity. Previously we have reported that Bcl-2 expression (another anti-apoptotic target of NF-κB) was decreased in aging [77]. These observations provide evidence for an important role and mechanisms by which decreased NF-κB sensitizes aging T cells to increased TNF-α-induced apoptosis. Our observations of decreased NF-κB activity in aged T cells is in agreement with those reported by Whisler et al [104] and Pahlvani and Harris [105]. Trebilcock and Ponnappan [106] demonstrated decreased induction of NF-κB in response to PMA and TNF-α. These authors further suggested that decreased induction of NF-κB could be due to decreased proteosome-mediated degradation of IκB [107]. In summary, it appears that decreased NF-κB activation contributes to the increased sensitivity of aged T cells to TNF-α-induced apoptosis.

## Naïve, Central Memory and Effector Memory T Cells

**Figure 5:**
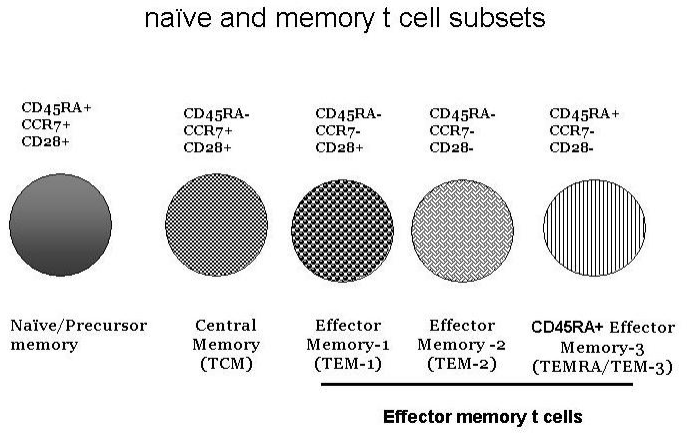
Phenotypically distinct five distinct subsets of CD8+ T cells, including naïve, central memory (TCM) and three type of effector memory (TEM-1, TEM-2, and TEM-3) cells.

Naïve T cells following exposure to a viral antigen undergo clonal expansion followed by clearance of virus. This phase is followed by a phase of contraction during which virus-specific T cells undergo apoptosis, and then number of virus-specific T cells stabilized and remained as memory T cells [108,109]. The memory T cells display differential expression of adhesion molecules (CD62L) and chemokine receptors (CCR-7), which allow them to home into lymph nodes and non-lymphoid tissue and mucosal sites, and to respond to microbes at peripheral tissue sites [110,111]. Therefore, CCR7+ and CD62^high ^T cells are found in lymph nodes, whereas CCR7- and CD62L^low ^are found in extranodal sites such as liver and lung [112,113]. Based upon these adhesion molecules and chemokine receptors, memory CD8+ T cells have been divided into "central memory" T cells for those that are found in lymphoid organs and "effector memory" T cells that are found in peripheral non-lymphoid tissues and mucosal sites [114-116]. These subpopulations of naïve, central and effector memory T cells are identified by a number of cell surface proteins [109,114-117]. Recently, we have further characterized these subsets of CD8+ T cells [118]. Naïve CD8+ T cells in addition to expression of CD45RA and CCR7 also express CD27 and CD28, whereas central memory (TCM) CD8+ T cells retain these cell surface antigens except CD45RA. Effector memory CD8+ T cells are further subdivided into three subsets. One subset of effector memory (TEM-1) is CCR7-CD45RA-CD28+, the second set of effector memory CD8+ T cells(TEM-2) is CCR7-CD45RA-CD28-), and the third set of effector memory CD8+ T cells (TEM-3/TEMRA) is CDCR7-CD45RA+ CD28-). Fig. 5 shows phenotypic characteristics of naïve and various memory CD8+ T cells in humans. Although generally it is considered that TEM-3/TEMRA subset is lacking from CD4+ T cells, we have observed a very small subset of TEM-3/TEMRA CD4+ T cells (1%), which is increased in aging (unpublished data). In analyzing data of Salusco et al [108], we also noticed a small population of TEM-3/TEMRA CD4+ T cells, which authors did not discuss in their results. During subsequent discussion, we will be using terminology TEM and TEMRA for two effector memory T cell subsets.

**Figure 6:**
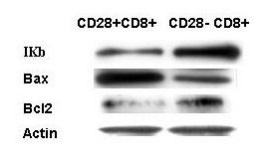
Expression of phosphorylated IKKβ, Bcl-2, and Bax in CD8+CD28+ and CD8+CD28- T cell lines. CD8+CD28- T cells, which are resistant to TNF-α-induced apoptosis, expressed increased levels of Bcl-2 and phospho IKKβ and decreased levels of Bax.

## Apoptosis in Naïve, Central Memory and Effector Memory CD8+ And CD4+ T Cells

### Death-receptor-induced apoptosis in naïve and memory CD4+ and CD8+ T cells

Recently, we have examined relative sensitivity of naïve and various memory CD8+ T cell subsets to TNF-α-induced apoptosis [83,119]. Mononuclear cells were activated with anti-CD3 monoclonal antibody for 2 days, cultured in an IL-2 containing medium for an additional three days and then activated with TNF-α. Our data show that naïve and TCM CD8+ T cells were sensitive whereas TEM and TEMRA CD8+ T cells were resistant to TNF-α-induced apoptosis. Apoptosis profile correlated with the activation of caspase-8 and caspase-3. However, no correlation was observed between relative sensitivity of four CD8+ T cell subsets to TNF-α-induced apoptosis and the expression of TNFR-I or TNFR-II. Therefore, we examined a role of downstream signaling events, including phosphorylation of IκB and NF-κB activity following activation with TNF-α and the expression of Bcl-2 and Bax in CD8+ T cell subsets. CD8+ CD28+ T cell line (containing naïve and TCM) and CD8+ CD28- T cell line (containing TEM and TEMRA were kindly provided by Dr. Abbe Vallejo, University of Pittsburg) were stimulated with TNF-α and IκB phosphorylation was measured by Western blotting, using IκB phospho antibodies and NF-κB activity was measured by ELISA-based assay. The expression of Bcl-2 and phosphorylated IκB and NF-κB activity were higher, whereas the expression of Bax was lower in TEM and TEMRA CD8+ T cells as compared to naïve and TCM CD8+ T cells (Figure 6). These data suggest that signaling molecules downstream of TNFRs may be responsible for differential sensitivity among subsets of CD8+ T cells to TNF-α-induced apoptosis. We have also observed that similar to CD8+ T cells, naïve and TCM CD4+ T cells (TCM> naïve) are sensitive to TNF-α-induced apoptosis, whereas TEM and TEMRA CD4+ T cells are resistant to TNF-α-induced apoptosis [120].

## Naïve, Central Memory and Effector Memory CD4+ And CD8+ T Cells in Aging

In aging, there is a significant reduction in naïve CD8+ T cells [76] and CD8+ CD28+ T cells, which contain both naïve and central memory CD8+ T cells [121]. In addition, there is an accumulation of CD8+CD28- T cells, which are oligoclonal and show characteristics of cellular senescence (i.e. short telomere length indicative of long replicative history), and increased IFN-γ production [122-127]. These CD8+ T CD28- cells are comprised of two subpopulations of effector memory CD8+ T cells [107], namely TEM and TEMRA CD8+ T cells. Our study shows a marked decrease in naïve and TCM CD8+ T cells and an increase in TEM and TEMRA CD8+ T cells [83]. Fagnoni et al [76] also observed an increase in primed CD8+CD28-CD45RA+ (equivalent to TEMRA) in aged humans.

## Apoptosis of Naïve, Central Memory and Effector Memory T Cell Subsets in Aging

### Activation-induced cell death (AICD)

Herndon et al [128] reported an increased AICD of naïve T (CD45RO-) T cells in aged humans and suggested its role in age-associated T cell deficiency. However, this study did not investigate apoptosis in memory T cells. Brezinska et al [121] have reported that AICD (as measured by DNA content and caspasese-3 activation) in CD8+CD28+ (containing naïve and TCM) and CD8+CD28- (containing TEM and TEMRA) was comparable between young and aging. However, data was presented from a single middle aged individual.

### CD95-mediated apoptosis

In our initial study, we observed that in aged humans, both CD45RA+ (naïve) and CD45RO+ (memory) CD4+ and CD8+ T cells were more sensitive to anti-CD95-induced apoptosis as compared to young subjects [77]. In addition, CD45RO+ displayed greater sensitivity to anti-CD95-induced apoptosis as compared to CD45RA+ CD4+ and CD8+ T cells in both young and aged subjects. Miyawaki et al (129) also reported that healthy adult memory T cells are more susceptible to anti-CD95-induced apoptosis as compared to naïve T cells. We reported decreased expression of Bcl-2 in both CD4+ and CD8+ T cells from aged humans as compared to young subjects; however, we did not examine Bcl-2 expression in naïve and memory subsets [77]. Shinohara et al [130] demonstrated decreased Bcl-2 expression in memory subsets of CD4+ and CD8+ T cells in healthy adults. This would be consistent with our observation of increased sensitivity of memory T cell subsets to death-receptor-mediated apoptosis as compared to naïve T cell subsets. Although a role of Bcl-2 family protein in death receptor pathway has been argued, several investigators have demonstrated that Bcl-2 blocks anti-CD95-induced apoptosis in mitogen-activated T cells [131,132]. Therefore, it is likely that decreased Bcl-2 expression in aging may play a role in increased sensitivity of T cell subsets in aged humans. Since CD45RA+ (contain naïve and TEMRA) and CD45RA-/CD45RO+ (contain TCM and TEM) are heterogenous and display differential sensitivity (naïve and TCM are sensitive and TEM and TEMRA are resistant) to other death stimuli, further studies are warranted with CD95-mediated signal in naïve and different memory subsets of CD8+ T cells.

### TNF-α-induced apoptosis

In our previous study we reported that both CD45RA+ naïve and CD45RA- memory CD4+ and CD8+ T cells from aged individuals were more sensitive to TNF-α-induced apoptosis [78]. Since CD45RA+ and CD45RA- T cells are heterogeneous we examined the relative sensitivity of naïve, TCM, TEM and TEMRA CD8+ and CD4+ T cell subsets to TNF-α-induced apoptosis in young and aged subjects. In aged humans, we observed that naïve and central memory CD8+ T cells displayed increased TNF-α-induced apoptosis as compared to young subjects, which is associated with increased caspase-8 and caspase-3 activation. Therefore, it appears that during aging decrease in naïve CD8+ T cells may be due to both decreased thymic output as well as increased apoptosis. We have also observed greater increased in apoptosis in TCM CD8+ T cells as compared to naïve CD8+ T cells in aged humans. In contrast, no significant difference was observed in the apoptosis of TEM and TEMRA CD8+ T cells between aged and young humans; both were comparably resistant to apoptosis [120]. This would suggest that the accumulation of TEM and TEMRA CD8+ T cells in aged humans is not due to changes in apoptosis and may be due to increased growth. We have observed that both TEM and TEMRA CD8+ T cells from young and aged subjects proliferate well in the presence of exogenous IL-2 and IL-15 even more than TCM CD8+ T cells (unpublished observation). We have also observed increased expression of IL-15 gene in CD8+ T cells from aged humans (by gene array) as compared to young subjects. These observations suggest that CD8+CD28- T cells generated by repeated activation *in vitro *are not a true model for CD8+CD28- T cells in aged humans since the latter cells do not proliferate (replicative senescence).

Since the expression of TNFR-I or TNFR-II is similar in young and aged humans, we have examined role of downstream signaling events in increased sensitivity of naïve and TCM CD8+ T cells in aged humans to TNF-α-induced apoptosis (manuscript in preparation). We have observed that CD28-CD8+ (containing naïve and TCM) from aged subjects display decreased phosphorylation of IKKα/β and IκB and decreased activation of NF-κB. Since NF-κB mediates its anti-apoptotic effect via induction of a number of anti-apoptotic molecules (IAP, FLIP, A20, Bcl-x_L_), we examined expression of these molecules by Western blotting. cIAP1, FLIPL, FLIPS, A20, and BCL-x_L _expression were decreased in aging CD28-CD8+ T cells. These data would suggest that decreased NF-κB activity may be central to increased sensitivity of naïve and TCM CD8+ T cells and perhaps of CD4+ T cells (since they also show similar profile of apoptosis in aging) to TNF-α-induced apoptosis.

## B Cells Subsets in Human Aging

B-lineage cells following immunoglobulin (Ig) gene rearrangement to generate functional antigen receptor are released into the peripheral blood B cell pool as naïve B cells. After exposure to a T-dependent antigen, Naïve be cells differentiate via one of two different pathways. They can either differentiate into short-lived Ig secreting cells or they migrate to germinal center, where high-affinity antigen-specific B cells are selected and undergo proliferation, somatic hypermutation of Ig V-region genes, isotype switching and develop into long-lived memory B cells [133-135]. Although a number of cell surface markers have been used to identify memory B cells including lack of surface IgD expression and expression of membrane IgG and IgA [135], or as IgD-CD38- B cells [136], these markers identify only certain populations of memory B cells. Recently, CD27 has been identified as a key marker of memory B cells and CD27 signaling promotes the differentiation of memory B cells to Immunoglobulin-secreting plasma cells [137].

Aging is associated with both quantitative and qualitative changes in humoral immunity. These include decreased levels of IgM and increased levels of IgG and IgA, decreased B cell repertoire, decreased primary and secondary specific antibody response to vaccine antigens and changes in antibody affinity [138]. It has been demonstrated that CD27 expression increases with age; lacking in cord blood B cells and approximately 40% of adult B cells express CD27 antigen [137]. We have examined the proportions and numbers of naïve and memory B cells in thirty young and fifty aged subjects. Our data show that the proportion of CD27+CD19+ memory B cells is significantly increased whereas the proportion of CD27-CD19+ naïve B cells is significantly decreased. This may explain reduced specific antibody response to novel antigens and increased accumulation of somatic mutation of Ig variable region genes in aged humans [139]. When B cells were analyzed for the expression of CD38 to define activated and switchable B cells, no significant difference was observed between young and aged subjects. Our observations are in complete contrast to recent report by Chong et al [74], who observed decreased memory and increased naïve B cells in aged subjects. The reason for this discrepancy is unclear. Our aged subjects were of middle socio-economical class, in good health and living independently. Since majority of seniors are on a number of supplements, including anti-oxidants and vitamin A and E, which can modify immune functions and apoptosis, our subjects were asked to discontinue all supplements at least one week prior to blood draw. Therefore, our population did not have any nutritional or chemical compounding factors. Chong et al [74] also demonstrated that naïve B cells were more resistant to spontaneous apoptosis as compared to memory B cells.

One small subpopulation of B cells express CD5 antigen, a 67 kDa monomer, which was originally identified as a subset of T cells. CD5+ B cells express a limited repertoire of V genes, secrete IgM antibodies that often react with self antigens (autoantibodies), and appear to be self-renewing population. These cells are expanded in autoimmune diseases. Since aging is associated with autoimmunity we have analyzed CD5+ B cells in aged subjects. We observed no difference in the proportions and numbers of CD5+ B cells between aged and young subjects. Furthermore, we examined the expression of CD95 and apoptosis in these subsets. We have observed increased proportions of CD95+CD5+ cells in aging as compared to young controls; however, the expression of CD95 did not correlate with apoptosis, which was comparable in young and aged subjects (manuscript submitted).

In summary, increased apoptosis in naive and TCM CD8+ T cells in aging appears to play an important role in lymphopenia of naïve and TCM CD8+ T cells (83), which might be responsible for decline in T cell functions and increased susceptibility to viral infection and increased frequency of cancer in aging. Data of B cells in aging is conflicting and more in-depth analysis is needed.

## References

[B1] Krammer PH (2000). CD95's deadly mission in the immune system. Nature.

[B2] Gupta S (2000). Suicidal journey in the Fas (t) track. Recent Res Dev Immunol.

[B3] Gupta S (2000). Molecular steps of cell suicide: An insight into immune senescence. J Clin Immunol.

[B4] Ashkanazi A, Dixit VM (1998). Death receptors: signaling and modulation. Science.

[B5] Gupta S (2000). Molecular steps of death receptor and mitocondrial pathways of apoptosis. Life Sci.

[B6] Gupta S (2001). Molecular steps of TNF receptor-mediated apoptosis. Curr Mol Med.

[B7] Gupta S (2002). Decision between life and death during TNF-induced signaling. J Clin Immunol.

[B8] Green DR, Evan GI (2002). A matter of Life and Death. Cancer Cell.

[B9] Kroemer G, Reed JC (2000). Mitochondrial control of cell death. Nature Med.

[B10] Martinou J-C, Green DR (2001). Breaking the mitochondrial barrier. Nature Rev Mol Cell Biol.

[B11] Zamzami N, Kroemer G (2001). The mitochondrion in apoptosis: how pandora's box opens. Nature Rev Mol Cell Biol.

[B12] Green DR, Droin N, Pinkoski M (2003). Activation-induced cell death in T cells. Immunol Rev.

[B13] Dhein J, Walczak H, Baumler C, Debatin KM, Kramer PH (1995). Autocrine T cell suicide mediated by APO-1/(fas-CD95). Nature.

[B14] Brunner T, Mogil RJ, LaFace D, Yoo NJ, Mahboubi A, Echeverri F, Martin SJ, Force WR, Lynch DH, Ware CF, Green DR (1995). Cell autonomous Fas (CD95)/Fas ligand interaction mediates activation-induced apoptosis in T-cell hybridomas. Nature.

[B15] Ju ST, Panka DJ, Cui H, Ettinger R, el-Khatib M, Sherr DH, Stanger BZ, Marshak-Rothstein A (1995). Fas (CD95/FasL interactions are required for programmed cell death after T cell activation. Nature.

[B16] Anderson MR, Tough TW, Davis-Smith T, Braddy S, Falk B, Schooley KA, Goodwin RG, Smith CA, Ramsdell F, Lynch DH (1995). Fas ligand mediates activation-induced cell death in human T lymphocytes. J Exp Med.

[B17] Mixter PF, Russell JQ, Budd RC (1994). Delayed kinetics of T lymphocyte anergy and deletion in lpr mice. J Autoimmunity.

[B18] Mogil RJ, Radvanyi L, Gonzalez-Quintial R, Miller R, Mills G, Theofilopoulos AN, Green DR (1995). Fas (CD95) participates in peripheral T cell deletion and associated apoptosis in vivo. Int Immunol.

[B19] Renno T, Hahne M, Tschopp J, MacDonald HR (1996). Peripheral T cells undergoing superantigen-induced apoptosis in vivo express B220 and upregulate Fas and Fas ligand. J Exp Med.

[B20] Scaffidi C, Fulda S, Srinivasan A, Friesen C, Li F, Tomaselli KJ, Debatin KM, Krammer PH, Peter ME (1998). Two CD95 (Apo-1/Fas) signaling pathways. EMBO J.

[B21] Li H, Zhu H, Xu C, Yuan J (1998). Cleavage of Bid by caspase-8 mediates mitochondrial damage in the Fas pathway of apoptosis. Cell.

[B22] Screaton G, Xu X-N (2000). T cell life and death signaling via TNF-receptor family members. Curr Opin Immunol.

[B23] Thomas B, Grell M, Pfizenmaier K, Scheurich P (1990). Identification of a 60-kDa tumor necrosis factor (TNF) receptor as the major signal transducing component in TNF responses. J Exp Med.

[B24] Darnay BG, Aggarwal BB (1997). Early events in TNF signaling: a story of associations and dissociations. J Leukocyte Biol.

[B25] Wallach D, Boldin M, Varfolomeev E, Beyaert R, Vandenabeele P, Fiers W (1997). Cell death induction by receptors of the TNF family: towards a molecular understanding. FEBS Lett.

[B26] Locksley RM, Kileen N, Lenardo MJ (2001). The TNF and TNF receptor superfamilies: interacting mammalian biology. Cell.

[B27] Beg AA, Baltimore D (1996). An essential role for NF-κB in preventing TNF-α-induced cell death. Science.

[B28] Ghosh S, May MJ, Kopp EB (1998). NF-κB and rel proteins: evolutionarily conserved mediators of immune responses. Annu Rev Immunol.

[B29] Baldwin AS (1996). The NF-κB and IκB proteins: new discoveries and insights. Annu Rev Immunol.

[B30] Karin M, Lin A (2002). NF-κB at the crossroads of life and death. Nature Immunol.

[B31] Ghosh S, Karin M (2002). Missing pieces in the NF-kB puzzle. Cell.

[B32] Micheau O, Tschopp J (2003). Induction of TNF receptor I-mediated apoptosis via two sequential signaling complexes. Cell.

[B33] Rothe M, Wong SC, Henzel WJ, Goeddel DV (1994). A novel family of putative signal transducers associated with the cytoplasmic domain of the 75 kDa tumor necrosis factor receptor. Cell.

[B34] Rothe M, Sarma V, Dixit VM, Goeddel DD (1995). TRAF2-mediated activation of NF-κB by TNF receptor 2 and CD40. Science.

[B35] Wajant H, Pfizenmaieer K, Scheurich P (2003). Tumor necrosis factor signaling. Cell Death Differ.

[B36] Maeda S, Chang L, Li ZW, Luo JL, Leffert H, Karin M (2003). IKKβ is required for prevention of apoptosis mediated by cell bound but not by circulating TNF-alpha. Immunity.

[B37] Hsu H, Huang J, Shu HB, Baichwal V, Goeddel DV (1996). TNF-dependent recruitment of the protein kinase RIP to the TNF receptor-1 signaling complex. Immunity.

[B38] Kelliher MA, Grimm S, Ishida Y, Kuo F, Stanger BZ, Leader P (1998). The death domain kinase RIP mediates the TNF-induced NF-κB signal. Immunity.

[B39] Ting AT, Pimentel-Muinos FX, Seed B (1996). RIP mediates tumor necrosis factor receptor 1 activation of NF-κB but not Fas/APO-1-initiated apoptosis. EMBO J.

[B40] Zha J, Zhou Q, Xu L-G, Chen D, Li L, Zhai Z, Shu H-B (2004). RIP5 is a RIP-homologous inducer of cell death. Biochem Biophys Res Comm.

[B41] McCarthy JV, Ni J, Dixit VM (1998). RIP-2 is a novel NF-κB activating and cell death-inducing kinase. J Biol Chem.

[B42] Sun X, Lee J, Navas T, Baldwin DT, Stewart TA, Dixit VM (1999). RIP3, a novel apoptosis-inducing kinase. J Biol Chem.

[B43] Chen L, Haider K, Mondo M, Cariappa A, Rowitch D, Pillai S (2001). Protein kinase C-associated kinase (PKK), a novel membrane associated ankyrin repeat-containing protein kinase. J Biol Chem.

[B44] Meylan E, Martinon F, Thome M, Gscwendt M, Tschopp J (2002). RIP4 (DICK/PKK), a novel member of RIP kinase family, activates NF-κB, and is processed during apoptosis. EMBO Rep.

[B45] Pahl HL (1999). Activators and target genes of Rel/NF-kB transcription factors. Oncogene.

[B46] Deveraux QL, Reed JC (1999). IAP family ptoteins: suppressor of apoptosis. Genes Dev.

[B47] Salvesen GS, Duckett CS (2004). IAP proteins: blocking the road to death's door. Nature Rev Mol Cell Biol.

[B48] Devereaux QL, Takahashi R, Salvesen GS, Reed JC (1997). X-linked IAP is a direct inhibitor of cell-death proteases. Nature.

[B49] Opipari AW, Hu HM, Yabkowitz R, Dixit VM (1992). The A20 zinc finger protein protects cells from tumor necrosis factor cytotoxicity. J Biol Chem.

[B50] Heyninck K, Beyaert R (2005). A20 inhibits NF-κB activation by dual ubiquitin-editing functions. Trends Biochem Sci.

[B51] Rothe M, Pan MG, Henzel WJ, Ayres TM, Goeddel DV (1995). The TNFR2-TRAF signaling complex contains two novel proteins related to baculoviral inhibitor of apoptosis proteins. Cell.

[B52] Micheau O, Lens S, Gaide O, Alevizopolous K, Tshopp J (2001). NF-κB signals induce the expression of c-FLIP. Mol Cell Biol.

[B53] Irmler M, Thome M, Hahne M, Schnieder P, Hoffmann K, Steiner V, Bodmer J-L, Schroter M, Burns K, Mattmann C, Rimoldi D, French LE, Tschopp J (1997). Inhibition of death receptor signals by cellular FLIP. Nature.

[B54] van Drogen F, O'Rourke SM, Stucke VM, Jaquenoud M, Neiman AM, Peter M (1997). The caspases-8 inhibitor FLIP promotes activation of NF-kappaB and ERK signaling pathways. Curr Biol.

[B55] Natoli G, Costanzo A, Ianni A, Templeton DJ, Woodgett JR, Balsano C, Levero M (1997). Activation of SAPK/JNK by TNF receptor 1 through a noncytotoxic TRAF-2-dependent pathway. Science.

[B56] Ichijo N, Nishida E, Irie K, Ten Dijke P, Saitoh M, Moriguchi T, Tagaki M, Matsumoto K, Miyazono K, Gotoh Y (1997). Induction of apoptosis by ASK1, a mammalian MAPKKK that activates SAPK/JNK and p38 signaling pathways. Science.

[B57] Vartfolmeev EE, Askenazi A (2004). Tumor necrosis factor: an apoptosis JuNKie?. Cell.

[B58] Deng Y, Ren X, Yang L, Lin Y, Wu X (2003). A JNK-dependent pathway is required for TNF-α-induced apoptosis. Cell.

[B59] De Smaele E, Zazzeroni F, Papa S, Nguyen DU, Jin R, Cong R, Franzoso G (2001). Induction of gadd45β by NF-κB downregulates proapoptotic JNK signaling. Nature.

[B60] Declercz W, Denecker G, Fiers W, Vandenabeele P (1998). Cooperation of both TNF receptors in inducing apoptosis: involvement of the TNF receptor-associated factor binding domain of the TNF receptor 75. J Immunol.

[B61] Haridas V, Darnay BG, Natrajan K, Helle R, Aggarwal BB (1998). Overexpression of the p80 TNFR leads to TNF-dependent apoptosis, nuclear factor-kappa B activation. J Immunol.

[B62] Weiss T, Grell M, Siekienski K, Muhlenbeck F, Durkop H, Pfizenmaier K, Scheurich P, Wajant H (1998). TNFR80-dependent enhancement of TNFR60-induced cell death is mediated by TNFR-associated factor 2 and is specific for TNFR60. J Immunol.

[B63] Chen FK, Lenardo MJ (2002). A crucial role for p80 TNF-R2 in amplifying p60 TNF-R1 apoptosis signals in T lymphocytes. Eur J Immunol.

[B64] Vandenabeele P, Declercq W, Vanhaesebroeck B, Grooten J, Fiers W (1995). Both TNF receptors are required for TNF-mediated induction of apoptosis in PC60 cells. J Immunol.

[B65] Tartaglia L, Pennica D, Goddel DV (1993). Ligand passing: the 75-kDa tumor necrosis factor (TNF) receptor recruits TNF for signaling by the p55-kDa TNF receptor. J Biol Chem.

[B66] Grell M, Zimmermann G, Gottfried E, Chen CM, Grunwald U, Huang DC, Wu Lee Y, Durkop H, Englemann H, Scheurich P (1999). Induction of cell death by tumor necrosis factor (TNF) receptor 2, CD40, and CD30: a role of TNFR1 activation by endogenous membrane-anchored TNF. EMBO J.

[B67] Gupta S (2003). Molecular signaling in death receptor and mitochondrial pathways of apoptosis. Internat J Oncol.

[B68] Hegde R, Srinivasula SM, Zhang Z, Wassell R, Mukattash R, Cilenti L, DuBois G, Lezebnik Y, Zervos AS, Fernandes-Alnemri T, Alnemri ES (2002). Identification of Omi/HtrA2 as a mitochondrial apoptotic serine protease that disrupts inhibitor of apoptosis protein-caspase interaction. J Biol Chem.

[B69] Suzuki Y, Imai Y, Nakayama H, Takahashi K, Takio T, Takahashi R (2001). A serine protease, HtrA2, is released from the mitochondria and interacts with XIAP, inducing cell death. Mol Cell.

[B70] Lorenzo HK, Susin SA, Penninger J, Kroemer G (1999). Apoptosis inducing factor (AIF): a physiologically old, caspases-independent effector of cell death. Cell Death Diff.

[B71] Loeffler M, Daugas E, Susin SA, Zamzani N, Metivier D, Nieminen A-L, Brothers G, Penninger JM, Kroemer G (2001). Dominant cell death induced by extra-mitochondrially targeted apoptosis-inducing factor. FASEB J.

[B72] Li LY, Luo X, Wang X (2001). Endonuclease G is an apoptotic DNAase when released from mitochondria. Nature.

[B73] Reed JC (1997). Double identity for protein of Bcl-2 family. Nature.

[B74] Chong Y, Ikematsu H, Yamaji K, Nishimura M, Nebishima S, Kashiwagi S, Hayashi J (2005). CD27+ (memory) B cell decrease and apoptosis- resistant CD27- (naïve) B cell increase in aged humans: Implications for age-related peripheral B cell developmental disturbance. Internat Immunol.

[B75] Gupta S (2002). Tumor necrosis factor-α-induced apoptosis in T cells from aged humans: a role of TNFR-I and downstream signaling molecules. Exp Gerontol.

[B76] Fagnoni FF, Vescovini R, Paserri G, Bologna G, Pedrazzoni M, Lavagetto G, Casti A, Franceschi C, Passeri M, Sansoni (2002). Shortage of circulating naïve CD8+ T cells provides new insights on immunodeficiency in aging. Blood.

[B77] Aggarwal S, Gupta S (1998). Increased apoptosis of T cell subsets in aging humans: Altered expression of Fas (CD95), Fas ligand, Bcl-2, and Bax. J Immunol.

[B78] Aggarwal S, Gollapudi S, Gupta S (1999). Increased TNF-α-induced apoptosis in lymphocytes from aged humans: changes in TNF-α receptor expression and activation of caspases. J Immunol.

[B79] Gupta S (2002). Tumor necrosis factor-α-induced apoptosis in T cell subsets from aged humans. Receptor expression and downstream signaling events. Exp Gerontology.

[B80] Gupta S, Chiplunkar S, Kim C, Yel L, Gollapudi S (2003). Effect of age on molecular signaling of TNF-α-induced apoptosis in human lymphocytes. Mech Ageing Dev.

[B81] Gupta S (2000). Molecular and biochemical pathways of apoptosis in lymphocytes from aged humans. Vaccine.

[B82] Gupta S (2003). A road to ruins: An insight into immunosenescence. Adv Cell Aging Gerontol.

[B83] Gupta S (2005). Molecular mechanisms of apoptosis in the cells of the immune system in human aging. Immunol Rev.

[B84] Phelouzat MA, Arbogast A, Laforge T, Quadri RA, Proust JJ (1996). Excessive apoptosis of mature T lymphocytes is a characteristic feature of human immune senescence. Mech Ageing Dev.

[B85] Phelouzat MA, Laforge T, Abrogast A, Quadri RA, Boutet S, Proust JJ (1997). Susceptibility to apoptosis of T lymphocytes from elderly humans is associated with increased in vivo expression of functional fas receptors. Mech Ageing Dev.

[B86] Lechner H, Amort M, Steger MM, Maczek C, Grubeck-Lobenstein B (1996). Regulation of CD95 (Apo-1) expression and the induction of apoptosis of human T cells: changes in old age. Int Arch Allergy Immunol.

[B87] Potestio M, Caruso C, Gervasi F, Scialabba G, D'Anna C, Di Lorenzo G, Balistreri CR, Candore G, Romano GC (1998). Apoptosis and aging. Mech Ageing Dev.

[B88] Dennett NS, Barcia RN, McLeod JD (2000). Age-associated decline in CD25 and CD28 expression correlate with an increased susceptibility to CD95 mediated apoptosis in T cells. Exp Gerontol.

[B89] Gupta S, Aggarwal S, Nguyen T (1998). Increased spontaneous apoptosis in T lymphocytes in DiGeorge anomaly. Clin Exp Immunol.

[B90] Aggarwal S, Gupta S (1999). Increased activity of caspase-3 and caspase-8 during Fas-mediated apoptosis in lymphocytes from aging humans. Clin Exp Immunol.

[B91] Zheng L, Fisher G, Miller RE, Peschon J, Lynch DH, Lenardo MJ (1995). Induction of apoptosis in mature T cells by tumor necrosis factor. Nature.

[B92] Fagiola U, Cossarizza A, Scala E, Fanales-Belasio E, Ortolani C, Cozzi E, Monti D, Franceschi C, Paganelli R (1993). Increased cytokine production in mononuclear cells of healthy elderly people. Eur J Immunol.

[B93] Brunnsgaard H, Andersen-Ranberg K, Hjelmborg JB, Pedersen BK, Jeu B (2003). Elevated tumor necrosis factor alpha and mortality in centenarians. Amer J Med.

[B94] Trzonkowski P, Myslizska J, Godlewska B, Szmit E, Lukaszuk K, Wieckiewicz J, Brydak L, Machala M, Landowski J, Mysliwski A (2004). Immune consequences of the spontaneous pro-inflammatory status in depressed elderly patients. Brain Behav Immun.

[B95] Njemini R, Demanet C, Mets T (2004). Inflammatory status as an important determinant of heat shock protein 70 serum concentration during aging. Biogerontology.

[B96] Penninx BWJH, Kritchevsky SB, Newman AB, Nicklas BJ, Simonsick EM, Rubin S, Nevitt M, Visser M, Harris T, Pahor M (2004). Inflammatory markers and incident mortality limitation in the elderly. J Amer Gerontol Soc.

[B97] De Maat MPM, Bladbjerg EM, Hjelmborg JVB, Bathum L, Jespersen J, Christensen K (2004). Genetic influence on inflammation variables in the elderly. Atheroscler Thromb Vasc Biol.

[B98] Kohut ML, Senchina DS, Madden KS, Martin AE, Felten DL, Moynihan JA (2004). Age effect on macrophage function vary by tissue site, nature of stimulant, and exercise behavior. Exp Gerontol.

[B99] Savioli S, Capri M, Scarcella E, Mangherini S, Franca I, Volterra V, De Ronchi D, Marini M, Bonafe M, Franceschi C, Monti D (2003). Age-dependent changes in the susceptibility to apoptosis of peripheral blood CD4+ and CD8+ T lymphocytes with virgin or memory phenotype. Mech Ageing Dev.

[B100] Aggarwal S, Gollapudi S, Yel L, Gupta AS, Gupta S (2000). TNF-α-induced apoptosis in neonatal lymphocytes: TNFRp55 expression and downstream pathways of apoptosis. Genes Immunity.

[B101] Gupta S, Kim C, Yel L, Gollapudi S (2004). A role of Fas-associated death domain (FADD) in increased apoptosis in aged humans. J Clin Immunol.

[B102] Gupta S, Bi R, Kim C, Yel L, Chiplunkar S, Gollapudi S (2005). A role of NF-κB signaling pathway in increased tumor necrosis factor-α-induced apoptosis of lymphocytes in aged humans. Cell Death Diff.

[B103] Gupta S (2004). A role of inhibitor of apoptosis (IAP) proteins in increased lymphocyte apoptosis in aged humans. Mech Ageing Dev.

[B104] Whisler RL, Beiqing L, Chen M (1996). Age-related decreases in IL-2 production by human T cells are associated with impaired activation of nuclear transcriptional factors AP-1 and NF-AT. Cell Immunol.

[B105] Pahlavani M, Harris MD (1996). The age-related changes in DNA binding activity of AP-1, NF-κB, and Oct-1 transcription factors in lymphocytes from rats. Age.

[B106] Trebilcock GU, Ponnappan U Evidence for lowered induction of nuclear factor kappa B in activated human T lymphocytes during aging. Gerontology.

[B107] Ponnappan U, Zhong M, Trebilcock GU (1999). Decreased proteosome-mediated degradation in T cells from the elderly: A role in immune senescence. Cell Immunol.

[B108] Sallusto F, Geginat J, Lanzavecchia A (2004). Central memory and effector memory T cell subsets: Function, generation, and maintenance. Ann Rev Immunol.

[B109] Kaech SM, Ahmed R (2001). Memory CD8+ T cell differentiation: initial antigen encounter triggers a developmental program in naïve cells. Nature Immunol.

[B110] Moser B, Loetscher P (2001). Lymphocyte traffic control by chemokines. Nature Immunol.

[B111] Schluns KS, Lefrancois L (2003). Cytokine control of memory T-cell development and survival. Nature Rev Immunol.

[B112] Kataoka T, Budd RC, Holler N, Thome M, Martinon F, Irmler M, Burns K, Masopust D, Vezys V, Marzo AL, Lanzavecchia A (2001). Preferential localization of effector memory cells in nonlymphoid tissue. Science.

[B113] Arbones SL, Ord DC, Ley K, Ratech H, Maynard-Curry C, Otten G, Capon DJ, Tedder TF (1994). Lymphocyte homing and leukocyte rolling and migration are impaired in L-selectin-deficienct mice. Immunity.

[B114] Campbell JJ, Bowman EP, Murphy K, Youngman KR, Siani MA, Thompson DA, Wu L, Zlotnik A, Butcher EC (1998). 6-C-kine (SLC), a lymphocyte adhesion-triggering chemokine expressed by high endothelium, is an agonist for MIP-13β receptor (CCR7). J Biol Chem.

[B115] Sallusto F, Lenig D, Forster R, Lipp M, Lanzavecchia A (1999). Two subsets of memory T lymphocytes with distinct homing potentials and effector functions. Nature.

[B116] Monteiro J, Baltiwala F, Ostere H, Gregersen PK (1996). Shortened telomere in clonally expanded CD28-CD8+ T cells imply a replicative history that is distinct from there CD28+CD8+ counterparts. J Immunol.

[B117] Weninger W, Crowley MA, Manjunath N, von Andriane UH (2001). Migratory properties of naïve, effector, and memory CD8 (+) T cells. J Exp Med.

[B118] Gupta S, Bi R, Su K, Yel L, Chiplunkar S, Gollapudi S (2004). Characterization of naïve, memory, and effector CD8+ T cells: Effect of age. Exp Gerontol.

[B119] Gupta S, Su H, Bi R, Gollapudi S Differential sensitivity of naïve and memory subsets of human CD8+ T cells to TNF-α-induced apoptosis.

[B120] Gupta S, Gollapudi S (2004). Molecular mechanisms of TNF-α-induced apoptosis in aging human T cell subsets. Int J Biochem Cell Biol.

[B121] Brzezinska A, Magalska A, Szybinska A, Sikora E (2004). Proliferation and apoptosis of human CD8+CD28+ and CD8+CD28- lymphocytes during aging. Exp Gerontol.

[B122] Posnett DN, Sinha R, Kabak S, Russo C (1994). Clonal populations of T cells in normal elderly humans: The cell equivalent to "benign monoclonal gammopathy". J Exp Med.

[B123] Effros RB, Boucher N, Porter V, Zhu X, Spaulding C, Walford RL, Kronenberg M, Cohen D, Schachter F (1994). Decline in CD28+ T cells in centenarians and in long-term T cell cultures: A possible cause of both in vivo and in vitro immunosenescence. Exp Gerontol.

[B124] Monteiro J, Baltiwala F, Ostrer H, Gregersen PK (1996). Shortened telomeres in clonally expanded CD28-CD8+ T cells imply a replicative history that is distinct from their CD28+CD8+ counterparts. J Immunol.

[B125] Bandres E, Merino J, Vazquez S, Inoges S, Moreno C, Subira ML, Sanchez-Ibarrola A (2000). The increase of IFN-γ production through aging correlates with the expanded CD8+CD28-CD57+ subpopulation. Clin Immunol.

[B126] Nociari MM, Telford W, Russo C (1999). Postthymic development of CD28-CD8+ T cell subset: age-associated expansion and shift from memory to naïve phenotype. J Immunol.

[B127] Saurwein-Teissl M, Lung TL, Marx F, Gschosser C, Asch E, Blasko I, Parson W, Bock G, Schonitzer D, Trannoy E, Grubeck-Loebenstein B (2002). Lack of antibody production following immunization in old age:Association with CD8+CD28- T cell clonal expansions and an imbalance in the production of Th1 and Th2 cytokines. J Immunol.

[B128] Herndon FJ, Hsu HC, Mountz JD (1997). Increased apoptosis of CD45RO- T cells with aging. Mech Ageing Dev.

[B129] Miyawaki T, Uehara T, Nabu R, Tsuji T, Yachie A, Yonehara Y, Taniguchi N (1992). Differential expression of apoptosis-related Fas antigen on lymphocyte subpopulations in human peripheral blood. J Immunol.

[B130] Shinohara S, Sawada T, Nishioka Y, Tohma S, Kisaki T, Inou T, Ando K, Ikeda M, Fuji H, Ito K Differential expression of Fas and Bcl-2 protein on CD+ T cells, CD8+ T cells and monocytes. Cell Immunol.

[B131] Iwai K, Miyawaki T, Takizawa T, Kondo A, Ohta K, Yachi A, Seki H, Taniguchi N (1994). Differential expression of b*cl-2 *and susceptibility to anti-Fas-mediated death in peripheral blood lymphocytes, monocytes and neutrophils. Blood.

[B132] Yoshino K, Kondo E, Cao L, Takahashi K, Hayashi K, Nomura S, Akagi T (1994). Inverse expression of Bcl-2 protein and Fas antigen in lymphoblasts in peripheral nodes and activated peripheral blood T and B lymphocytes.

[B133] Liu Y-J, Oldsfield S, MacLennan IC (1988). Memory B cells in T cell-dependent antibody responses colonize the splenic marginal zones. Eur J Immunol.

[B134] Smith KG, Hewitson TD, Nossal GJ, Tarlinton DM (1996). The phenotype and fate of the antibody-forming cells of the splenic foci. Eur J Immunol.

[B135] Liu Y-J, Banchereau J (1996). The paths and molecular control of peripheral B cell development. Immunologist.

[B136] Liu Y-J, Barthelemy C, de Boutetler O, Arpin C, Durand I, Banchereau J (1995). Memory B cells from human tonsils colonize mucosal epithelium and directly present antigen to T cells by rapid upregulation of B7-1 and B7-2. Immunity.

[B137] Agematsu K, Hokibara S, Nagumo H, Komiyama A (2000). CD27: a memory B cell marker. Immunology Today.

[B138] Ghia P, Melchers F, Rolink AG (2000). Age-dependent changes in B lymphocyte development in man and mouse. Exp Gerontol.

[B139] Banerjee M, Mehr R, Belelovsky A, Spencer J, Walters D (2000). Age-and tissue-specific differences in human germinal center B cell selection revealed by analysis of IgV_H _gene hypermutation and linage tree. Eur J Immunol.

